# Effects of extracellular polymeric substances on the aggregation of *Aphanizomenon flos-aquae* under increasing temperature

**DOI:** 10.3389/fmicb.2022.971433

**Published:** 2022-09-08

**Authors:** Dailan Deng, Han Meng, You Ma, Yongqi Guo, Zixuan Wang, Huan He, Jin-e Liu, Limin Zhang

**Affiliations:** ^1^Jiangsu Center for Collaborative Innovation in Geographical Information Resource Development and Application, School of Environment, Nanjing Normal University, Nanjing, China; ^2^Jiangsu Engineering Lab of Water and Soil Eco-Remediation, Nanjing, China; ^3^Green Economy Development Institute, Nanjing University of Finance and Economics, Nanjing, China

**Keywords:** cyanobacterial bloom, temperature, *Aphanizomenon flos-aquae*, aggregation, extracellular polymeric substances

## Abstract

Aphanizomenon flos-aquae (*A. flos-aquae*) blooms are serious environmental and ecological problems. Extracellular polymeric substances (EPSs) are among the most important indicators for the growth and aggregation of *A. flos-aquae*. In this study, the secretion of the EPS matrix under temperature rise (7–37°C) was investigated and the role of this matrix in *A. flos-aquae* aggregation was quantified. First, when the temperature increased, the aggregation ratio increased from 41.85 to 91.04%. Meanwhile, we found that when soluble EPSs (S-EPSs), loosely bound EPSs (LB-EPSs), and tightly bound EPSs (TB-EPSs) were removed successively, the aggregation ratio decreased from 69.29 to 67.45%, 61.47%, and 41.14%, respectively. Second, the content of polysaccharides in the EPS matrix was higher than the content of proteins under temperature change. The polysaccharide in TB-EPSs was closely related to the aggregation ability of *A. flos-aquae* (*P* < 0.01). Third, PARAFAC analysis detected two humic-like substances and one protein-like substance in EPSs. Furthermore, Fourier transforms infrared spectroscopy (FTIR) showed that with increasing temperature, the polysaccharide-related functional groups increased, and the absolute value of the zeta potential decreased. In conclusion, these results indicated that a large number of polysaccharides in TB-EPSs were secreted under increasing temperature, and the polysaccharide-related functional groups increased correspondingly, which reduced the electrostatic repulsion between algal cells, leading to the destruction of the stability of the dispersion system, and then the occurrence of aggregation. This helps us to understand the process of filamentous cyanobacterial aggregation in lakes.

## Introduction

Seasonal algal blooms are increasingly threatening ecosystem functions and human health in Lake, with *Microcystis* predominating in summer and *Aphanizomenon flos-aquae* (*A. flos-aquae*) predominating in spring and autumn, while cyanobacteria often show seasonal replacement in Lake (Tang et al., 2019; Weber et al., 2020). Seasonal variations are reflected in temperature changes, which directly or indirectly affect the growth rate, reproduction rate, and enzyme activity of algal cells by affecting photosynthesis and respiration (Yang et al., 2020). More importantly, a large area of *A. flos-aquae* has appeared in some lakes during the summer in recent years. Many lakes and reservoirs have *A. flos-aquae* blooms, including Lake Dianchi in China (Jin et al., 2022), Lake Biwa in Japan (Yamamoto, 2009), the Baltic Sea in Europe (Laamanen et al., 2002), and Lake Ontario in Hamilton Harbour (Zastepa and Chemali, 2021), with severe eutrophic problems. *A. flos-aquae* is a toxigenic filamentous cyanobacterium that exhibits increased growth at high temperatures and remains active at low temperatures with high density (Yamamoto, 2009; Jin et al., 2022).

Aside from the direct effects on cyanobacterial density, higher temperatures increase nutrient diffusion to the cell surface, which may favor cyanobacterial growth (Peperzak, 2003). The external environment affects algal cells, and self-regulation changes the secretion of proteins, polysaccharides, lipids, and other extracellular polymeric substances (EPSs) to adapt to the environment (Brennan and Owende, 2010; Scott et al., 2010; Schlesinger et al., 2012). EPSs thus play an important role in relieving external stress on algal cells in the absence of additional energy input. In some algal species, EPSs are viscous polymers composed of polysaccharides, proteins, and other substances produced during cell growth that contribute to cell aggregation (Sheng et al., 2010). Because of the presence of multiple functional groups, the cyanobacterial EPS matrix can consistently bind to the surface of colloidal particles, influencing their stability/aggregation propensity (Lin et al., 2016; Si et al., 2019). Algae typically aggregate and migrate to the water surface, causing blooms (Ramesh et al., 2006), and filamentous cyanobacteria are gaining attention. This prompted us to investigate whether *A. flos-aquae* aggregation is related to EPSs.

Extracellular polymeric substances were divided into three fractions soluble EPS (SL-EPS), loosely bound EPS (LB-EPS), and tightly bound EPS (TB-EPS) (Xu et al., 2013a). The majority of organic matter is distributed in TB-EPS, with a minor portion distributed in S-EPS and LB-EPS (Xu et al., 2013b). Polysaccharides are mostly found in TB-EPS, while proteins are mostly found in S-EPS and LB-EPS. Takeda et al. (1992) investigated the EPS aggregation mechanism of *Nocardia amarae* and discovered that proteins were highly active. Allen et al. (2004) discovered that *Thauera* sp. aggregation in a sewage system was closely related to polysaccharides in EPSs. These disparate results prompted us to consider which specific components of the EPS matrix had the greatest influence on the aggregation of *A. flos-aquae*. To the best of our knowledge, however, this information has not been elucidated.

Therefore, to reveal the bloom formation mechanism of *A. flos-aquae*, it is necessary to study its aggregation characteristics. In this study, by combining physical and chemical analyses, the fluorescence excitation-emission matrix (EEM), Fourier transform infrared spectroscopy (FTIR), and the zeta potential by simulating seasonal changes under increasing temperatures, the effects of the release of EPSs on the aggregation ability of *A. flos-aquae* under temperature changes were investigated. The aggregation rate and the content of the components of EPSs were examined for correlations. The findings contribute to a better understanding of filamentous cyanobacterial aggregation behavior and provide theoretical support for cyanobacteria control in lakes.

## Materials and methods

### Cultivation of *Aphanizomenon flos-aquae*

The cyanobacterial strain *Aphanizomenon flos-aquae* FACHB-1039, which was isolated from cyanobacterial blooms, was provided by the Institute of Hydrobiology, Chinese Academy of Sciences, and it was cultured in batch mode in conical flasks. Exponentially growing *A. flos-aquae* was inoculated into the BG-11 medium an initial absorbance of 0.1 and cultured under a light: dark cycle of 12:12 at a light intensity of 30 μmol photons m^–2^ s^–1^. Growth was monitored by measuring the absorbance of *A. flos-aquae* cultures at 680 nm using a spectrophotometer (Tecan SPARK, Austria). To simulate a temperature change, the culture temperature was increased by 2°C every 3 days from 7 to 37°C before being cultured at 7°C for a week. Algal samples were collected on the last day of every temperature setting for aggregation potential measurement and EPS extraction. All flasks were shaken three times per day at regular intervals during the experimental period. Each treatment was conducted in triplicate.

### Determination of the aggregation potential

The aggregation ability of the algal cells is usually evaluated by the aggregation rate (Tan et al., 2018). The aggregation rate was determined according to Xu et al. (2014). At 680 nm, the optical density of the *A. flos-aquae* sample was determined (A_0_). After 6 h of standing in a clean test tube, the algal liquid from 2 cm of the upper layer was carefully removed and the optical density was measured again at 680 nm (A_t_). The aggregation ratio of the *A. flos-aquae* samples was calculated by using the following equation:


$$\mathrm{Aggregation}\,\mathrm{ratio}=\left(1-\frac{A_{\text{t}}}{A_{0}}\right)\times 100$$

### Extracellular polymeric substance extraction and spectral analysis

Extracellular polymeric substances consist of S-EPS, LB-EPS, and TB-EPS (Li and Yang, 2007; Sheng et al., 2010; Xu et al., 2010). In this study, EPS extraction was performed according to Xu et al. (2013a).

The three-dimensional excitation-emission matrix fluorescence spectrum (3D-EEM) was measured using a fluorescence spectrometer (LF-1301009, Thermo Fisher, United States) and the measurement was carried out in accordance with Xu et al. (2013b). The difference is that the emission (Em) and excitation (Ex) wavelengths range from 250 to 450 nm at 5 nm increments and from 200 to 550 nm at 10 nm increments, respectively. Other Rayleigh and Raman scattering data were filtered using interpolation adopted from Bahram et al. (2006). PARAFAC modeling was carried out for all EEM spectra of the EPS samples collected over the whole growth period (Yamashita and Jaffé, 2008). PARAFAC analysis was conducted in Matlab 2016b (Mathworks, Natick, MA, United States) using the DOMFluor toolbox.^1^ No outliers were found by leverage comparison, and a total of 48 fluorescence EEM data arrays (48 samples × 21 Ex × 21 Em) were obtained for the PARAFAC models, and 2–7 components were computed. The residual analysis was conducted according to Stedmon and Bro (2008).

Extracellular polymeric substance extractions were completely dried to powder in a freeze dryer. After freeze-drying, the EPS samples and dried KBr were mixed at a ratio of 1:100 and homogenized in an agate grinder. In total, 150 mg of the mixture was compressed and analyzed using a spectrum FTIR spectrometer (Nexus 670, Nicolet). The scanning conditions were as follows: a spectral range of 4000–400 cm^–1^, 32 scans and a resolution of 4 cm^–1^.

### Zeta potential and contact angle measurement

The zeta potential represents the electrostatic force on the cell surface, and it can be used to evaluate the stability of dispersions (Tan et al., 2020). The zeta potential was measured at 25°C using a Zeta sizer (Nano ZS90, Malvern Instruments Ltd., United Kingdom), according to Hadjoudja et al. (2010). This method was carried out using Doppler electrophoresis, which is based on the relative displacement of charged particles in an applied electric field. A 50 μL sample was taken and diluted 100 times with PBS solution before the test. Three copies of each measurement were taken.

Contact angle measurement (θ) was performed by using a contact angle analyzer (JC2000D, Powereach, Shanghai, China), according to the standard sessile drop method (Van Oss, 1995). Pure water was dropped onto the membrane containing *A. flos-aquae*. The arithmetic means of at least seven independent measurements were used to calculate all contact angle values.

### Statistical analysis and chemical measurement

All chemicals used in this work were of analytical grade. Data are presented as the means ± standard deviations. Significant differences were analyzed by one-way analysis of variance (ANOVA). SPSS 22.0 was used for all statistical analyses. Pearson correlation was performed to describe the relationships among the EPS content, aggregation ability and algal growth.

Using glucose as a standard, the polysaccharide content was determined using the phenol-sulfuric acid method (Dubois et al., 1956). Protein contents were determined according to Bradford (1976) with bovine serum albumin as a standard. Cell density was measured at an absorbance of 680 nm. Furthermore, maximum photoelectron production (Fv/Fm) and actual photoelectron production (Fv′/Fm′) were measured using a pulse amplitude-modulated fluorometer (AquaPen, PSI, Czechia).

## Results

### Growth and aggregation of *Aphanizomenon flos-aquae*

**FIGURE 1:**
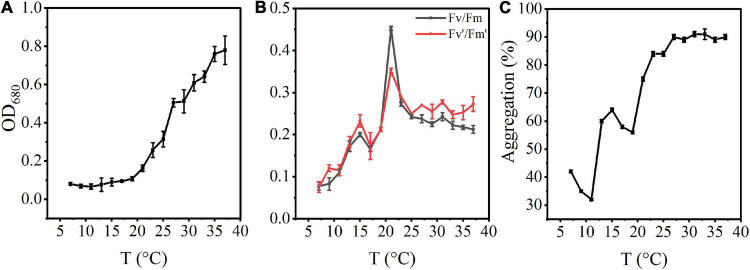
Effects of rising temperature on the growth of *A. flos-aquae* and aggregation with temperature. **(A)** Variation of algal density with temperature; **(B)** Variation of photosynthetic activity with temperature; **(C)** Variation of aggregation ratio with temperature.

The density of *A. flos-aquae* generally showed an increasing trend with increasing temperature (Figure 1A). At low temperature, the algal density was low, and it recovered at about 15°C, changed significantly at 20°C, and reached a stable growth state at 35°C.

The photosynthetic activity first increased and then decreased with increasing temperature (Figure 1B). Fv/Fm increased gradually from 7 to 21°C, and reached a maximum value of 0.45 at 21°C. At this temperature, the specific growth rate also reached a maximum value, indicating that the optimal growth temperature was 21°C.

The aggregation ratio of *A. flos-aquae* gradually increased from the initial 41.85–91.04% with increasing temperature (Figure 1C). At 7–11°C, the aggregation rate gradually decreased. From 11 to 27°C, aggregation increased with increasing temperature. It reached the maximum value at 31°C (91.04%) and then tended to a stable state, indicating that increasing temperature promoted aggregation of *A. flos-aquae*. Many factors influence the aggregation, including the surface properties of algal cells, which have a direct impact on the aggregation of the cells (Xu et al., 2016).

### Physicochemical properties of the extracellular polymeric substance matrix and effects of extracellular polymeric substance extraction on aggregation variations

Under increasing temperature, the polysaccharide content continued to increase and reached a stable state at 33°C. Among EPSs, the polysaccharide TB-EPS fraction exhibited a significant increase during the whole process, showing a significant correlation with the growth and aggregation of *A. flos-aquae* (Table 1). Additionally, the ratio of polysaccharides in EPSs changed. For example, the proportion of polysaccharides in the TB-EPS component increased from an initial 8% (3.05 mg/L out of 37.04 mg/L) to 77% (21.72 mg/L out of 28.19 mg/L) at 31°C. Similarly, the ratio of proteins in the EPSs also changed. As shown, the content of polysaccharides in the EPS matrix was higher than that of proteins, regardless of the temperature differences. Furthermore, it was clear that high temperature (29–37°C) induced a greater release of EPSs compared with low temperature (7–15°C).

**TABLE 1:** Pearson correlation analysis of the aggregation, algal density, zeta potential, and protein and polysaccharide in EPS components.

		Aggregation		OD_680_		Zeta potential^3^	
				
		R^1^	P^2^	R^1^	P^2^	R^1^	P^2^
S-EPS	Protein	–0.016	0.953	–0.020	0.941	–0.320	0.440
	Polysaccharide	0.328	0.216	0.121	0.654	0.104	0.807
LB-EPS	Protein	0.428	0.098	0.230	0.392	0.169	0.690
	Polysaccharide	0.662**	0.005	0.701**	0.002	0.824*	0.012
TB-EPS	Protein	0.512*	0.043	0.492	0.192	0.693	0.056
	Polysaccharide	0.757**	0.001	0.949**	0.000	0.778*	0.023

^1^R stands for Pearson correlation coefficient. ^2^P represents the significance level. ^3^Zeta potential analysis data used charge changes of different EPS fractions. *Correlation is significant at 0.05 level (two-tailed); **Correlation is significant at the 0.01 level (two-tailed).

**FIGURE 2:**
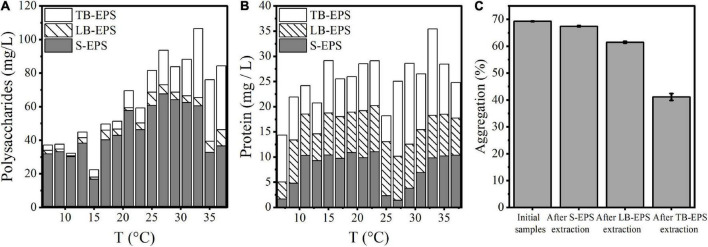
Variations of composition in EPS matrix with temperature and the relationship between aggregation and EPS fractions for *A. flos-aquae*. **(A)** Variation of polysaccharides with temperature; **(B)** Variation of protein with temperature; **(C)** Variation of aggregation with removed EPS fraction in turn.

The changes in the aggregation ratio during the extraction of EPSs at the optimum growth temperature of *A. flos-aquae* showed that when the EPS matrix was extracted, the aggregation ratio of *A. flos-aquae* decreased (Figure 2C). This finding indicated that the EPS matrix played a more important role in *A. flos-aquae* aggregates. Among EPSs, the aggregation ratio after extraction of TB-EPS decreased significantly, which may be related to the substance composition of TB-EPS.

### PARAFAC of excitation-emission matrix spectra

**FIGURE 3:**
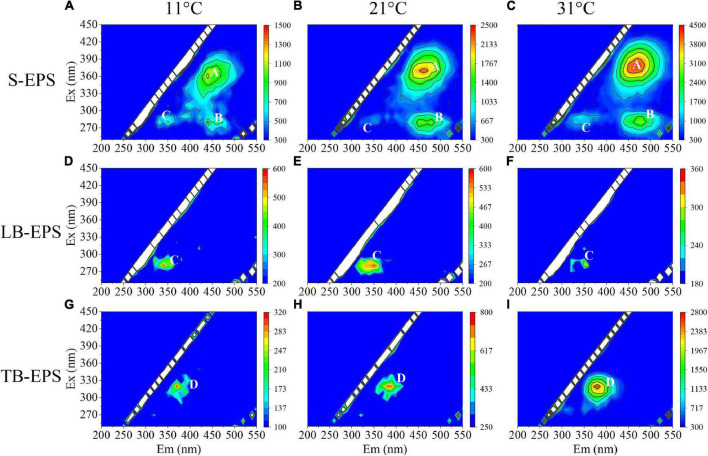
EEM spectra of EPS fractions of *A. flos-aquae* at different temperatures (peak A and peak B standed for humic-like, peak C and peak D standed protein). **(A–C)** Represented the EEM diagram of S-EPS at 11, 21, and 31°C; **(D–F)** represented the EEM diagram of LB-EPS at 11, 21, and 31°C; **(G–I)** represented the EEM diagram of TB-EPS at 11, 21, and 31°C.

The EEM spectra of EPSs at different temperatures showed that the fluorescence intensity changed significantly with the increase in temperature, but the organic composition in the EPS matrix did not change. Specifically, S-EPS exhibited two humic-like substances (peak A and peak B) and one protein-like substances (peak C), while both LB-EPS (peak C) and TB-EPS (peak D) exhibited a protein-like substances (Figure 3). The results of the EEM contours demonstrated that protein-like substances were detected in all fractions of EPSs, whereas humic-like substances were mainly distributed in the S-EPS fraction.

**FIGURE 4:**
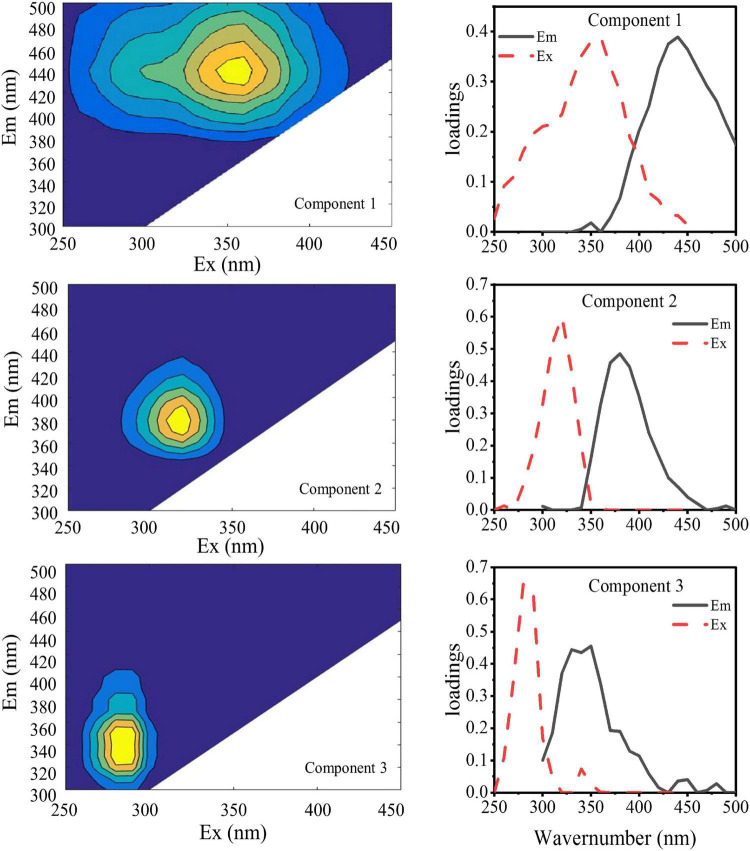
EEM contours, emission (solid lines) and excitation (dotted lines) loading of the three components identified by the DOMFluor-PARAFAC analysis for EPS produced by *A. flos-aquae* (Component 1, Component 2, and Component 3 were obtained by PARAFAC of 48 fluorescence EEM data arrays).

**FIGURE 5:**
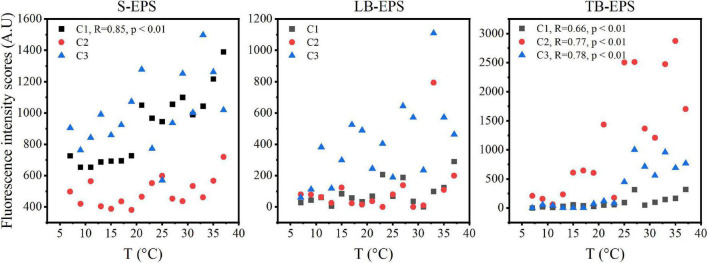
The scatter plots of fluorescence intensity scores in different EPS fractions with increasing temperature of *A. flos-aquae* (C1 and C2 defined as humic-like substances, C3 defined as tryptophan-like substances; R stands for Pearson correlation coefficient; *p* represents the significance level).

The DOMFluor-PARAFAC results indicated the Ex/Em loading of the three components (Figure 4). The fluorescence spectra revealed three components: a protein-like component and two humic-like components. Component 1 (C1) and Component 2 (C2) were defined as humic-like substances, with C1 having a much wider Ex spectrum than C2. Component 3 (C3), which exhibited a single Ex/Em peak of 280/350 nm, was identified as a tryptophan-like substance (Xu et al., 2013a). The proportion of the fluorescence intensity of the three components (C1–C3) in the three fractions of EPSs differed under temperature changes (Figure 4). Although EEM-PRAFAC analysis has been used for the characterization of EPSs, it has not been applied to investigate *A. flos-aquae* growth to date. The scatter plots of the PARAFAC derived component scores are shown in Figure 5. C3 had the highest fluorescence intensity scores in the S-EPS fraction, followed by C1 and C2. In the TB-EPS fraction, however, C2 had the highest fluorescence intensity scores, followed by C3 and C1. C3 had the highest fluorescence intensity scores in the LB-EPS fraction, at low temperatures, C2 > C1, and with increasing temperatures, C1 > C2. The fluorescence intensity scores of C1 and C2 in all fractions of EPSs, as well as of C3 in the S-EPS and TB-EPS fractions, increased as the temperature increased. Humic-like substances in the S-EPS fraction were positively correlated with *A. flos-aquae* growth, whereas tryptophan-like and humic-like substances were associated with *A. flos-aquae* growth in the TB-EPS fraction.

### Fourier transforms infrared spectroscopy spectra of extracellular polymeric substance and *Aphanizomenon flos-aquae*

**FIGURE 6:**
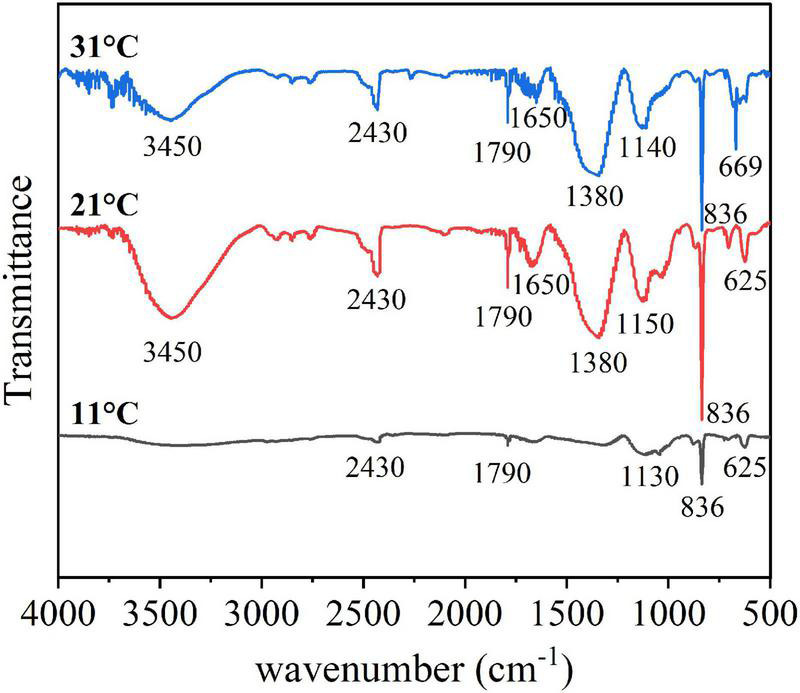
FTIR spectra of the EPS in different temperature.

The characteristic peaks of the FTIR spectra of EPSs varied significantly at different temperatures, mainly in the part of the functional groups related to polysaccharides (Figure 6). Specifically, the broad band at 3450 cm^–1^ was attributed to the stretching vibrations of both O-H (of polysaccharides) and N-H (of proteins), the band at 2430 cm^–1^ was attributed to -COO- stretching vibrations (Ueshima et al., 2008), and the band at 1790 cm^–1^ was attributed to C = O stretching vibrations (Song et al., 2014). Proteins are associated with the amide I (1700–1600 cm^–1^), amide II (1600–1500 cm^–1^), and amide III (1300–1200 cm^–1^) ranges (Ueshima et al., 2008; Wang et al., 2012; Fang et al., 2019), while polysaccharides and nucleic acids are associated with the 1200–900 cm^–1^ range (Yuan et al., 2011). Thus, bands attributed to the C-O-C ring and C-O stretching vibrations at 1150–1140 cm^–1^ were derived from polysaccharides (Yuan et al., 2011; Fang et al., 2019). These functional groups of EPS are closely related to the chemical properties (Yu et al., 2012; Zhu et al., 2012). In this study, the comparison of the functional groups changes at different temperatures showed that EPSs had obvious spectral changes in the band range of 3450 cm^–1^ and 1200–900 cm^–1^ at different temperatures. Polysaccharide synthesis was inhibited at low temperature, resulting in fewer polysaccharide-related functional groups. As the temperature increased, the algal cells secreted more polysaccharides, and more functional groups were discovered.

### Zeta potential and contact angle

The surface properties of algal cells, which are primarily determined by surface charge and hydrophilicity, influence cell aggregation. The measured contact angle and zeta potential data are shown in Table 2. The higher the absolute value of the zeta potential, the stronger the electrostatic repulsion on the surface of the algae and the less likely they are to aggregate. As shown, from 7 to 35°C, the zeta potential of the algal surface decreased from –15.29 ± 1.297 mV to –17.43 ± 1.19 mV and then increased to –13.77 ± 1.60 mV. The absolute value of the zeta potential increased first and then decreased with the increase in temperature. This indicated that the higher the temperature, the less negative the charge on the surface, the lower the repulsion between the cells, and the greater the aggregation ability. The lower surface electronegativity of *A. flos-aquae* made it more conducive to aggregation. This trend was consistent with the change in the aggregation rate. In addition, the change of the zeta potential was related to polysaccharides in LB-EPS and TB-EPS (Table 1).

**TABLE 2:** Changes of water contact angle and zeta potential under different temperature.

Temperature (°C)	Contact angle (°)	Zeta potential (mV)
11	37.49 ± 1.08	–17.43 ± 1.19
15	54.80 ± 1.43	–16.17 ± 1.15
19	51.05 ± 1.73	–16.39 ± 1.46
23	54.46 ± 0.70	–14.11 ± 1.62
27	44.54 ± 1.09	–14.05 ± 1.46
31	42.93 ± 1.56	–13.78 ± 1.60
35	43.74 ± 1.55	–13.77 ± 1.60

The contact angle between samples and water can characterize hydrophobicity. The results showed that *A. flos-aquae* at all experimental temperatures was characterized as being hydrophilic (θ_*H_2_ O*_θ_*H2O*_ ≤ 90°), and the algae were the least hydrophobic at 15°C, and the hydrophilicity gradually increased with the increase in temperature.

## Discussion

### Effects of temperature on growth and aggregation of *Aphanizomenon flos-aquae*

Cyanobacteria have a competitive advantage due to their higher growth rate at higher temperatures (Paerl and Huisman, 2008). Due to the increase in algal cell accumulation and growth rate, the biomass of *A. flos-aquae* in our increasing temperature experiment showed an increasing tendency as the temperature increased from 11 to 35°C (Figure 1A). This is consistent with the research findings under the influence of temperature on cyanobacterial biomass in Lake Taihu (Yang et al., 2020). Although rising temperatures promote cyanobacterial growth, high temperatures do not promote the formation of large colonies (Zhu et al., 2016), and temperatures that are too high can result in damage to proteins, DNA, and lipids and modulation of membrane stability in algal cells (Babele et al., 2017). Our simulation experiments also revealed a decreasing trend in photosynthetic activity and growth rate as the temperature gradient increased to 21 and 31°C, respectively (Figure 1B). Thus, 20–30°C may be the optimum growth temperature for *A. flos-aquae*.

### Effects of temperature the release of EPS from *Aphanizomenon flos-aquae*

Beyond the direct effects on cyanobacterial growth rates, higher temperatures increase nutrient diffusion toward the cell surface which may be beneficial to cyanobacteria (Peperzak, 2003). EPSs are distributed on the surface of algal cells and are mainly composed of proteins and polysaccharides (Richert et al., 2005). Therefore, the quantitative study of extracellular polysaccharides and proteins during the rising temperature process revealed the same results as Vogel (1995). The concentration of extracellular protein and polysaccharide increased gradually with increasing temperature (Figure 2), indicating that the higher temperature promoted the release of organic matter by algae cells to the external environment.

### The relationship between extracellular polymeric substance and aggregation

Extracellular polymeric substances have been reported to increase the aggregation ability of algal cells to adapt to the external environment (Su and Yu, 2005; Yang et al., 2012). Therefore, this study explored the relationship between the EPS matrix and *A. flos-aquae* aggregation (Figure 2C) and discovered that the aggregation ratio gradually decreased after the removal of S-EPS, LB-EPS, and TB-EPS, indicating that EPSs play a significant role in *A. flos-aquae* aggregation, which is consistent with the results reported by Xu et al. (2014). A variety of biomacromolecules in EPSs has been reported to have high activity, such as proteins produced by *Nocardia amarae* (Takeda et al., 1992), polysaccharides from *Thauera* sp. (Allen et al., 2004), and glycoprotein from *Arcuadendron* sp. (Lee et al., 1995). In this study, it was found that the polysaccharide activity in TB-EPS was higher, which was closely related to the aggregation of *A. flos-aquae* (*P* < 0.01). The composition and principal functional groups of the EPS matrix were further studied by 3D-EEM and FTIR, and it was found that organic matter in TB-EPS was related to the growth of *A. flos-aquae* and the polysaccharide functional groups dominated (Figure 6). This is in agreement with the results obtained by Xu et al. (2014). However, Xu et al. (2014) found that each fraction of EPS exhibited two fluorescence peaks, which is different from the results of this study. In addition, compared with the peak locations reported previously (Sheng and Yu, 2006; Ji et al., 2021), the location peaks in this study showed shifts. On the one hand, this may be due to the specificity of *A. flos-aquae.* On the other hand, it may be caused by differences in the extraction methods used for EPSs. This study also measured the zeta potential of *A. flos-aquae*, and it was discovered that the surface charge of algal cells changed with temperature, which may be related to the composition of EPSs. As a result of these changes, the aggregation of *A. flos-aquae* changed with temperature.

## Conclusion

In this study, the EPSs of *A. flos-aquae* were divided into three fractions, S-EPS, LB-EPS, and TB-EPS, to investigate the effect of EPSs on the aggregation process of *A. flos-aquae* as the temperature increased. The results showed that TB-EPS might play an important role in the formation of aggregates of *A. flos-aquae.* Polysaccharides in TB-EPS, in particular, contributed significantly to algal cell aggregation. With multiple methods, we concluded that as the temperature increased, many polysaccharides were synthesized and released into the extracellular environment, reducing the electrostatic repulsion between cells and promoting cell aggregation. This work provides basic information on the aggregation of *A. flos-aquae*, which helps us to understand the process of filamentous cyanobacterial aggregation in lakes.

## Data availability statement

The original contributions presented in this study are included in the article/supplementary material, further inquiries can be directed to the corresponding authors.

## Author contributions

DD: investigation, methodology, data analysis, and writing – original draft. HM: investigation, methodology, conceptualization, data analysis, and writing – review and editing. YM: investigation, methodology, and data analysis. YG and ZW: methodology and investigation. HH: supervision and writing – review and editing. J-eL: methodology, investigation, conceptualization, supervision, and writing – review and editing. LZ: methodology, investigation, conceptualization, supervision, and writing – review and editing. All authors contributed to the article and approved the submitted version.

## Footnotes

1
http://www.models.life.ku.dk/
